# Effect of Pluronic P103 Concentration on the Simple Synthesis of Ag and Au Nanoparticles and Their Application in Anatase-TiO_2_ Decoration for Its Use in Photocatalysis

**DOI:** 10.3390/molecules27010127

**Published:** 2021-12-26

**Authors:** Frida Karem Rivas-Moreno, Adan Luna-Flores, Daniel Cruz-González, Valeria Jordana González-Coronel, Manuel Sánchez-Cantú, José Luis Rodríguez-López, Uriel Caudillo-Flores, Nancy Tepale

**Affiliations:** 1 Facultad de Ingeniería Química, Benemérita Universidad Autónoma de Puebla, Avenida San Claudio y 18 Sur, Puebla 72570, Mexico; frida.rivas@correo.buap.mx (F.K.R.-M.); adan.luna@correo.buap.mx (A.L.-F.); daniel.cruz@correo.buap.mx (D.C.-G.); valeria.gonzalez@correo.buap.mx (V.J.G.-C.); manuel.sanchez@correo.buap.mx (M.S.-C.); 2 Advanced Materials Department, Instituto Potosino de Investigación Científica y Tecnológica A.C., Camino a la Presa de San José 2055, Lomas 4 Sección, San Luis Potosi 78216, Mexico; jlrdz@ipicyt.edu.mx; 3 Centro de Nanociencias y Nanotecnología, Universidad Nacional Autónoma de México, Ensenada 22860, Mexico; ucaudillo@gmail.com

**Keywords:** green synthesis, photocatalysis, gold nanoparticles, silver nanoparticles, triblock copolymer, soft templates, TiO_2_

## Abstract

Silver and gold nanoparticles were synthesized under environmentally-friendly reaction conditions by using a biodegradable copolymer and water as a solvent. The triblock copolymer Pluronic P103 was utilized as a stabilizing agent or soft template to produce Ag and Au nanoparticles (NPs) of different sizes. Moreover, in the synthesis of Au NPs, the polymer acted as a reducing agent, decreasing the number of reagents used and consequently the residues produced, hence, rendering the procedure less complicated. It was observed that as the concentration of the polymer increased, the size of the metallic NPs augmented as well. However, AgNPs and AuNPs prepared with 1 and 10 wt% Pluronic P103, respectively, showed a significant decrease in particle size due to the presence of polymeric soft templates. The hybrid materials (metal/polymer) were characterized by UV-Vis spectroscopy, DLS, and TEM. The pre-synthesized nanoparticles were employed to decorate anatase-TiO_2_, and the composites were characterized by DRS, XRD, BET surface area measurements, the TEM technique with the EDS spectrum, and XPS spectroscopy to demonstrate NPs superficial incorporation. Finally, methylene blue was used as a probe molecule to evidence the effect of NPs decoration in its photocatalytic degradation. The results showed that the presence of the NPs positively affected methylene blue degradation, achieving 96% and 97% removal by utilizing TAg0.1 and TAu10, respectively, in comparison to bare anatase-TiO_2_ (77%).

## 1. Introduction

The concept of green chemistry, i.e., green manufacturing, green production, and clean chemistry, sustainable chemistry, was formally established by Paul Anastas and John Warner in the 1990s [1]. It was defined as the “design of chemical products and processes to reduce or eliminate the use and generation of hazardous substances” [1,2,3], and it can be applied in all stages of the product’s life cycle, e.g., design, manufacture, and final disposal [4,5].

Therefore, NPs synthesis through simple methods has acquired great importance. For instance, triblock copolymers in aqueous solutions have been widely used, since they act as reductants and stabilizers. In consequence, they decrease the number of elements involved in a reaction [6]. Recently, triblock copolymer Pluronic P103 has been employed in AgNPs and AuNPs synthesis due to its hydrophobic character, commercial availability, and biocompatibility [7,8]. However, this polymer is very versatile, and a change in temperature and concentration results in new and attractive arrangements. Therefore, it is a material with great potential in the synthesis of NPs.

On the other hand, catalysis is one of the most versatile tools of green chemistry, in that it reduces waste generation and the energy required for transformations while increasing synthesis selectivity [2,9]. In heterogeneous photocatalysis, semiconductors are widely used in advanced oxidation processes, such as environmental remediation of waste-water, due to (a) the ability to generate charge carriers when exposed to radiation at a determined wavelength [10], (b) their electronic structure [11], and (c) their light absorption properties [12]. The most used materials in heterogeneous photocatalysis are semiconductor oxides, e.g., TiO_2_, ZnO, and SnO_2_ [13], due to their high oxidizing properties and stability, together with their low cost and toxicity [12,14]. Although TiO_2_ has gained considerable attention in photocatalytic applications [13,15,16,17,18,19], it is essential to improve its performance [12,14,20]. Its main disadvantages are rapid recombination of electron-hole pairs and the bandgap values (anatase phase: 3.2 eV, rutile phase: 3.0 eV), which limits its ultraviolet absorption (λ < 390 nm) [19,21].

Consequently, several strategies have been developed to improve TiO_2_ visible light activity, including doping [11], decoration [12,22,23,24], and doping/decoration [25]. The semiconductor/metallic junction results in an efficient electron trap that prevents the recombination of electron-hole pairs due to the Schottky junction [26]. However, the amount of heterojunctions could affect the transfer of such photoelectrons; thus, it is essential to possess a proper relationship [27]. Metal NPs improve the photocatalytic performance due to localized surface plasmon resonance (LSPR) [23,24], which contributes to increased radiation absorption and the excitation of active charge carriers [26,28,29]. Therefore, the surface modification of TiO_2_ with metal NPs improves its photocatalytic properties, extending its activation range from UV to UV-Visible radiation, which is advantageous considering that the activation source can be solar energy [12,13,28].

Among the most common metals in nanometer sizes that present LSPR we find Ag, Au, Pd, and Pt [17]. In addition, both Ag and Au have antibacterial, anticancer, fungicidal, and catalytical properties [12,14,17,19,30,31,32,33,34]. Gao et al. [22] synthesized Ag- and Au-decorated TiO_2_ membranes by two methods: hydrothermal synthesis and photo-reduction. These authors showed that metallic NPs enhanced the photo-response of the semiconductor in the visible light region, improving its photocatalytic properties in rhodamine B degradation. In addition, they observed that the LSPR effect of AgNPs was stronger than that of AuNPs. Narkburekeau et al. [29] degraded rhodamine B using anatase-phase TiO_2_ with AgNPs deposited on its surface by the chemical reduction method followed by a calcination process, which led to an increase in crystallinity and photocatalytic efficiency. Ismail et al. [35] reported a simple synthesis method through the photo-deposition of precious metals onto mesoporous TiO_2_ networks utilizing the F127 triblock copolymer as a template. The authors demonstrated that the precious metals/TiO_2_ nanocomposites were more photoactive than bare TiO_2_. Liu et al. [36] presented a simple synthetic method to prepare Au/TiO_2_ nanocomposite materials. These authors used the amphiphilic block copolymer PEO-b-PS dissolved in tetrahydrofuran (THF) as a co-template to produce AuNPs with specific sizes. The nanocomposites demonstrated significantly higher activity in photocatalytic methanol dehydrogenation.

To our recent knowledge, there are few reports in the literature investigating the photocatalytic characteristics of Ag/TiO_2_ or Au/TiO_2_ systems using triblock copolymers in a simple synthesis.

Herein, the preparation of a green photocatalyst, i.e., TiO_2_ decorated with either AgNPs or AuNPs, is reported. The composites were prepared in a simple and environmentally friendly manner. To start, NPs were synthesized using different concentrations of the predominantly hydrophobic and biodegradable triblock copolymer Pluronic P103 in an aqueous solution. This procedure has a relevant effect on particle size. In the synthesis of AgNPs, NaBH_4_ was utilized as the reducing agent. Therefore, the amount of polymer employed in their fabrication was different from that used in AuNPs synthesis, where only the polymer was employed. Later, commercial TiO_2_ was decorated with NPs followed by calcination at 500 °C. The new composites were characterized by DRS, XRD, and BET surface area measurements, TEM and EDS, and XPS spectroscopy. The photocatalytic performance of this system was evaluated under ultraviolet and visible-light irradiation using MB dye, which usually is taken as a representative organic-pollutant molecule, and compared against the behavior of bare calcined TiO_2_. It was shown that decoration of the TiO_2_ surface with NPs increases the photocatalytic efficiency.

## 2. Results and Discussion

### 2.1. Silver Nanoparticles (AgNPs)

**Figure 1:**
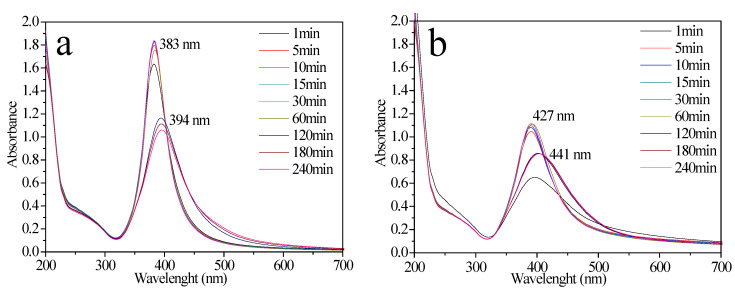
UV-Vis spectra of the AgNPs using NaBH_4_: (**a**) AgNPs P103 0% and (**b**) AgNPs P103 0.1%, both synthesized at 30 °C.

The optical properties of NPs such as size, shape, concentration, and agglomeration state, can be inferred by UV-Vis spectroscopy. Figure 1a displays the UV-Vis spectra of the sample prepared in the absence of the copolymer (AgNPs P103 0%). As observed, the spectra exhibit absorption bands between 383 and 394 nm, which are related to the LSPR of spherical AgNPs with a radius below 20 nm [8,37,38,39]. An increase in the absorption intensity is observed at the early stages. Santos et al. [38] proposed that intensity is related to concentration; consequently, higher intensity means higher concentration. Nevertheless, intensity later decreases, and the bands present a redshift, suggesting the presence of larger particles, indicating that the NPs probably agglomerated due to electrostatic interactions between them [38]. Figure 1b shows the UV-Vis spectra of AgNPs using an aqueous solution of Pluronic P103 at 0.1 wt% (AgNPs P103 0.1%). The polymer modifies absorption-band behavior, where less intensity and a redshift are observed. Moreover, band broadening occurred. In solution, the Pluronic P103 structure changes with increasing concentration, i.e., monomers-micelles-agglomerates of micelles [8,40] that modify AgNPs formation.

**Figure 2:**
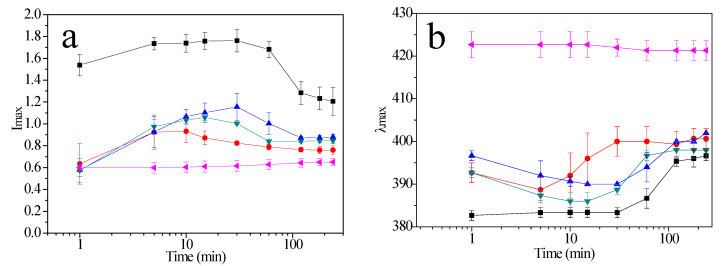
Semilog-plot of (**a**) I*_max_* and (**b**) λ*_max_* as a function of reaction time for AgNPs synthesized at 30 °C and different Pluronic P103 concentrations (wt%): (■) AgNPs P103 0%; (●) AgNPs P103 0.01%; (▲) AgNPs P103 0.1%; (▼) AgNPs P103 0.5%, and (◂) AgNPs P103 1%. Solid lines are aids to the eye. Error bars indicate standard deviation for triplicate measurements.

The kinetics of AgNPs formation was evaluated using maximum plasmon resonance absorbance (I*_max_*) and wavelength at the absorption peak (λ*_max_*). Figure 2a presents I*_max_* vs. time. For AgNPs P103 0%, it can be appreciated that intensity decreases after 60 min which is assigned to a size increase [37]. AgNPs synthesized in aqueous solutions of Pluronic P103 at 0.01, 0.1, and 0.5 wt% exhibited similar behavior with I*_max_* changing over time. As shown in Figure 1b for AgNPs P103 0.1%, the polymer decreases the intensity of absorbance and promotes band broadening, denoting an increase in both nanoparticle size and polydispersity [37]. However, for aqueous solutions of Pluronic P103 at 1 wt%, I*_max_* remains nearly constant during the reaction. Recently, a versatile and inexpensive method was developed to produce oval-shaped micelles with the capacity to act as a soft template or nanoreactor where small sized AgNPs were formed [8]. Thus, it is proposed that at 1 wt% of P103, surface cavities of the soft template allow an orderly process, causing I*_max_* to remain constant.

On the other hand, the λ*_max_* position is related to the size and shape of NPs [38,41]. Figure 2b shows that for AgNPs P103 0%, λ*_max_* remains constant for 30 min, showing an increase of around 60 min, which is related to a size enlargement [42,43]. It finally reached a constant value. However, λ*_max_* behaves differently when the polymer is used. This takes place for AgNPs P103 0.01%, AgNPs P103 0.1%, and AgNPs P103 0.5%. During the first stage, the wavelength decreases, with all samples showing a blueshift, revealing a process in which the newly formed particles show a decomposition into smaller ones [44]. Later, the blueshift becomes a redshift, indicating an increase in particle size [8,45]. Finally, after 100 min, λ*_max_* remains constant. On the other side, for AgNPs P103 1%, λ*_max_* exhibits plateau stability throughout the reaction, suggesting that soft templates control NPs growth [37]. An increase in polymer concentration enhances λ*_max_*. Different authors have reported that higher amounts of polymer produce larger NPs because polymers form part of the nanostructure [43,46,47].

**Figure 3:**
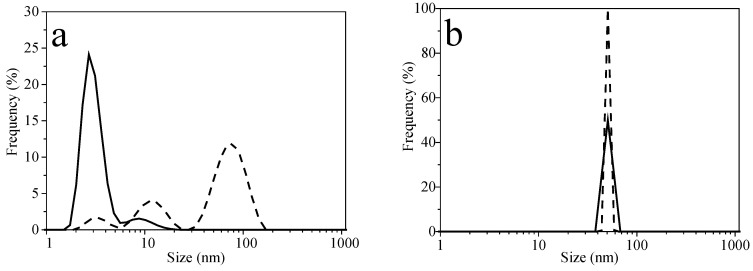
DLS plots for size distribution profiles of AgNPs synthesized at 30 °C: (**a**) AgNPs P103 0% and (**b**) AgNPs P103 0.1%. Intensity size distribution (dashed line). Volume size distribution (continuous line).

Now, to obtain a general idea of the structural changes of AgNPs, DLS was used. DLS is an excellent tool for micelles with core and swollen corona [48]. Figure 3 exhibits the particle-size distribution of (a) AgNPs P103 0% and (b) AgNPs P103 0.1%. The intensity size distribution of AgNPs P103 0% is trimodal: 3, 12, and 79 nm. However, the volume size distribution exhibits only two peaks (3 and 9 nm), showing a greater frequency of 3-nm particles. For AgNPs P103 0.1%, there is only one signal, both in intensity and volume, indicating the presence NPs of 50 nm. This response demonstrates that the polymer used during the synthesis of NPs greatly affects their size.

**Figure 4:**
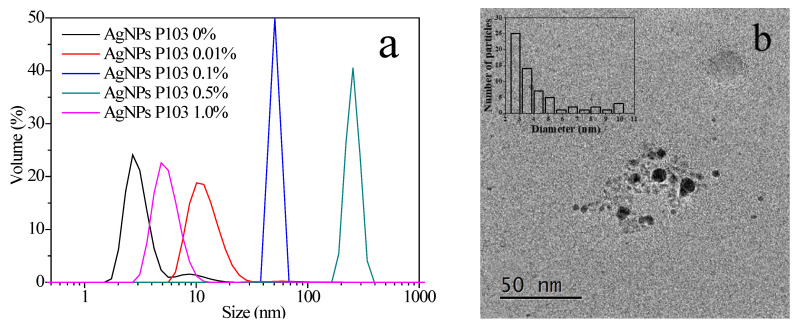
(**a**) DLS plot for size distribution profiles of AgNPs synthesized at 30 °C with and without Pluronic P103 at different concentrations (wt%). (**b**) TEM for AgNPs P103 1%.

The increase in volume size distribution is evident when the polymer concentration is augmented (0.01, 0.1, and 0.5 wt%), as observed in Figure 4a. The presence of a single peak may indicate large AgNPs or AgNPs agglomeration [49]. This behavior correlates with UV-Vis spectroscopy (Figure 2b), in that an increase in polymer concentration promotes a redshift, indicating an increase in particle size [38,42]. However, for AgNPs P103 1%, the size decreases, and only one peak is observed near 6 nm. The intensity and width are close to that of AgNPs P103 0%, suggesting the formation of smaller nanostructures. TEM shows AgNPs arranged on the surface of a soft template, with a size of 4.2 ± 2.0 nm (Figure 4b).

### 2.2. Gold Nanoparticles (AuNPs)

In the synthesis of AuNPs, Pluronic P103 acts as a reducing and stabilizing agent, which comprises an advantage over AgNPs synthesis. It is considered a green synthesis, in that it uses few reagents, is affordable, and is practical. AuNPs’ synthesis employing different concentrations of Pluronic P103 (0.4–5 mM) has been extensively studied because it is possible to modulate NPs size (8–30 nm) [6,50]. However, our group recently synthesized AuNPs on the surface of soft P103 templates (10–20 wt%), reporting the formation of tiny NPs (3-nm in size) [7] that due to their size, are very promising in the area of catalysis.

**Figure 5:**
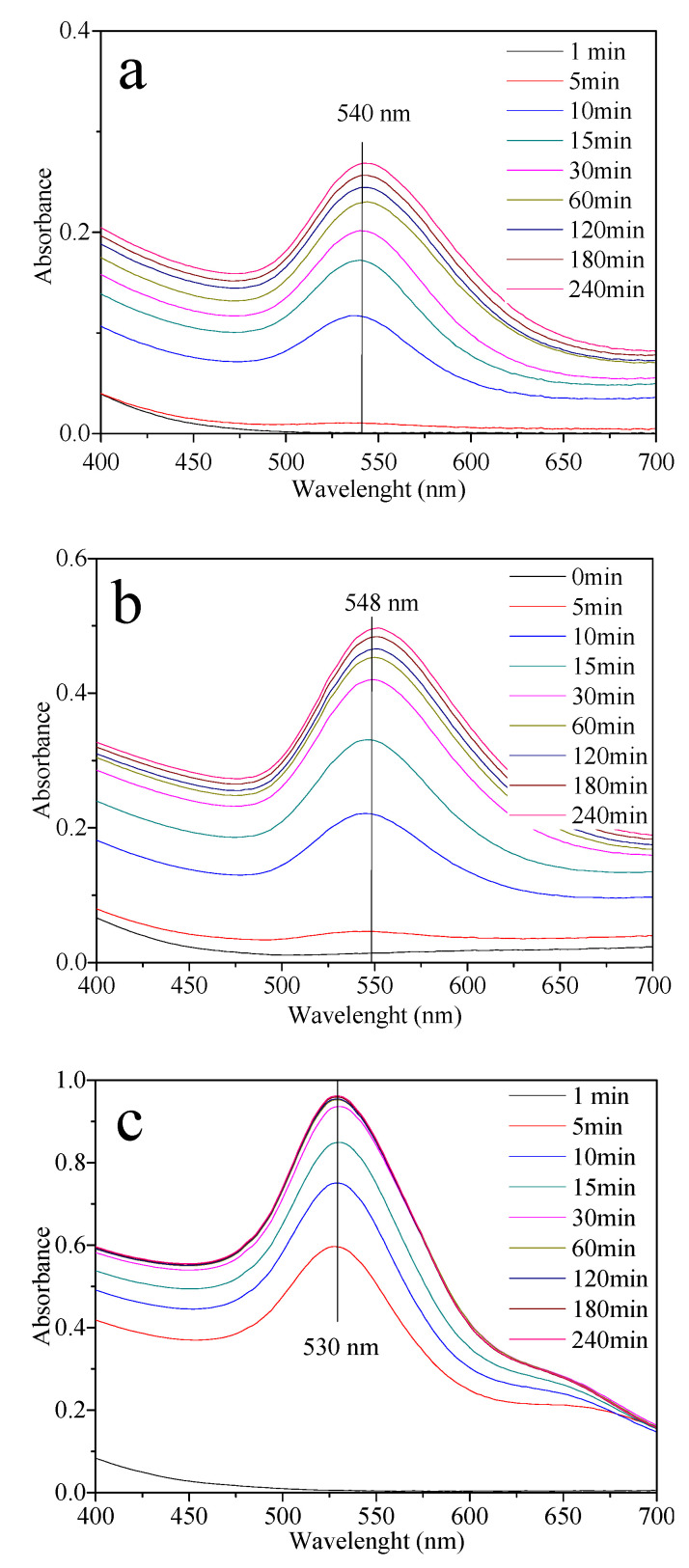
UV-Vis spectra of AuNPs using only Pluronic P103: (**a**) AuNPs P103 0.5%; (**b**) AuNPs P103 1%, and (**c**) AuNPs P103 10%, all synthesized at 30 °C.

The absorption spectra of AuNPs utilizing Pluronic P103 aqueous solutions at different concentrations are displayed in Figure 5. Figure 5a shows the synthesis of AuNPs using Pluronic P103 at 0.5 wt%. A plasmon peak is evident at 540 nm, inferring the presence of spherical nanoparticles [47]. An increase in polymer concentration (1.0 wt%) leads to higher absorbance and a redshift (Figure 5b). The broad plasmonic band indicates that AuNPs exhibit a large size distribution or aggregation, or both. Surprisingly, an excess of polymer (10 wt%) shifts the plasmon position to a lower wavelength (530 nm), suggesting the presence of smaller NPs (Figure 5c). The SPR band becomes less broad and more symmetric, indicating a narrow size distribution or less aggregation [49].

**Figure 6:**
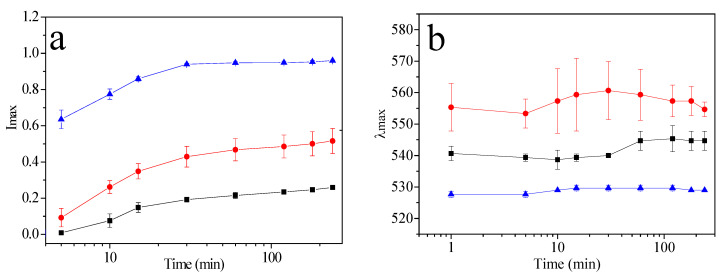
Semilog-plot of (**a**) I*_max_* and (**b**) λ*_max_* as a function of reaction time for AuNPs synthesized at 30 °C and different Pluronic P103 concentrations (wt%): (■) AuNPs P103 0.5%; (●) AuNPs P103 1%, and (▲) AuNPs P103 10%. Solid lines are aids to the eye. Error bars indicate standard deviation for triplicate measurements.

Figure 6a reveals I*_max_* increases in all three colloidal solutions. It was reported that increasing the copolymer concentration of the number of produced NPs is favored [47]. Concerning λ*_max_* (Figure 6b), AuNPs P103 1% increases from 553 to 560 nm, with a considerable standard deviation throughout the reaction. Micellization dynamics could be responsible for this behavior, due to the structural changes of micelles taking place in two different ways: (1) insertion of free copolymers into existing micelles, and (2) melt-fragmentation or insertion-expulsion [51]. On the other hand, λ*_max_* for AuNPs P103 10% reveals a nearly constant wavelength at 530 nm, with a low standard deviation attributed to higher stability in the formation of smaller NPs [52].

**Figure 7:**
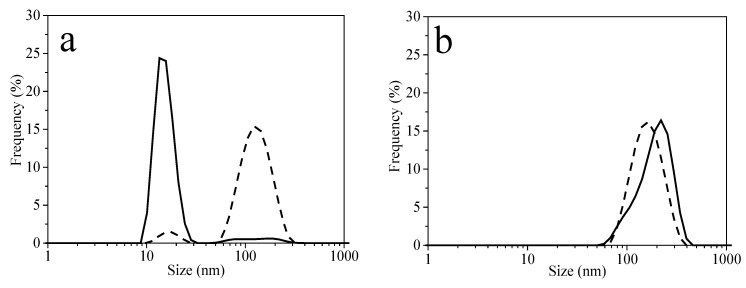

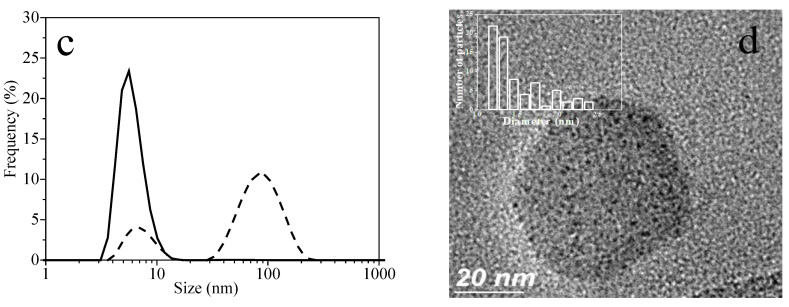
DLS plots for size distribution profiles of AuNPs synthesized at 30 °C with Pluronic P103 at different concentrations (wt%): (**a**) AuNPs P103 0.5%; (**b**) AuNPs P103 1%, and (**c**) AuNPs P103 10%. Intensity size distribution (dashed line), volume size distribution (continuous line). (**d**) TEM for AuNPs P103 10%.

Figure 7 displays the nanoparticle size distribution plots. For AuNPs P103 0.5%, two peaks (15 and 125 nm) are identified in intensity size distribution, suggesting the presence of NPs and NP agglomerates. However, a predominant peak (15 nm) in the volume size distribution is common (Figure 7a). In the case of AuNPs P103 1%, a single peak (~150 nm) with a broad size distribution is presented (Figure 7b). DLS measurements corroborated the observations made by UV-Vis spectroscopy (λ*_max_* AuNPs P103 1% > λ*_max_* AuNPs P103 0.5%, Figure 6b). For AuNPs P103 10%, two signals are obtained in intensity size distribution, i.e., 6 and 90 nm. The former signal can be attributed to isolated NPs, and the latter, to hybrid micelles or soft templates, in which very small size AuNPs are trapped. Regarding volume size distribution, a predominant signal is obtained at 6 nm (Figure 7c). From the TEM micrographs, it is observed that tiny AuNPs are arranged on the surface of a soft template with a size of 1.5 ± 0.35 nm (Figure 7d). As can be observed in Figure 7a,c, both have similar behaviors; however, when analyzing the UV-Vis spectra, λ*_max_* is different (see Figure 6b), which highlights that the polymer concentration determines the size of the NPs [7]. As a reference, other researchers, such as Chatterjee and Hazra [49], synthesized 4-nm AuNPs entrapped in Pluronic P123 polymeric templates. Also, Antonisamy et al. [53] incorporated tiny AuNPs on the surface of polymeric templates formed with the Pluronic F127 copolymer.

**Figure 8:**
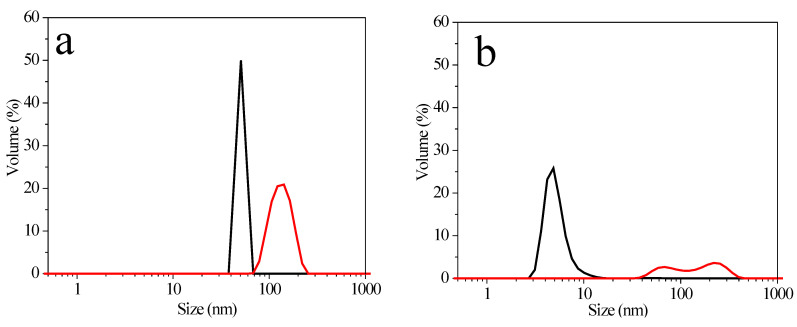

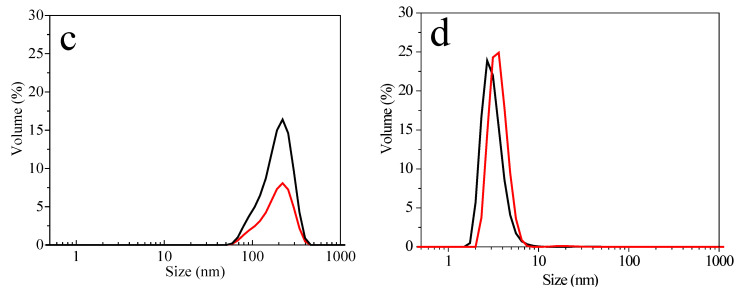
Size distribution profiles: (**a**) AgNPs P103 0.1%; (**b**) AgNPs P103 1%; (**c**) AuNPs P103 1%, and (**d**) AuNPs P103 10%. Before (black line) and after (red line) washing and centrifugation.

Figure 8 shows silver and gold NPs sizes before and after washing and centrifugation. AgNPs P103 0.1% (Figure 8a) present two sizes, 50 and 142 nm, before and after washing, respectively. AgNPs P103 1% (Figure 8b) have a size of 8 nm prior to washing, and two distributions, i.e., 68 and 220 nm, after washing. The increase in size infers the agglomeration of the particles and is attributed to the extreme centrifugation conditions. On the other hand, the colloidal solution of AuNPs P103 1% (Figure 8c) presents a single size distribution at 220 nm before and after washing. Similarly, the colloidal solution of AuNPs P103 10% (Figure 8d) exhibits a minimal change in the size of NPs, suggesting that the polymeric soft template prevents interaction between the NPs, avoiding their agglomeration [7].

### 2.3. Characterization of TiO_2_-AgNPs and TiO_2_-AuNPs

The centrifuged NPs were used to decorate the TiO_2_ surface. The decoration technique decreases the probability of the pair electron-hole recombination of the photo-excited electrons that are transferred from the conduction band to the NPs deposited on the TiO_2_ surface [12,23,24].

**Figure 9:**
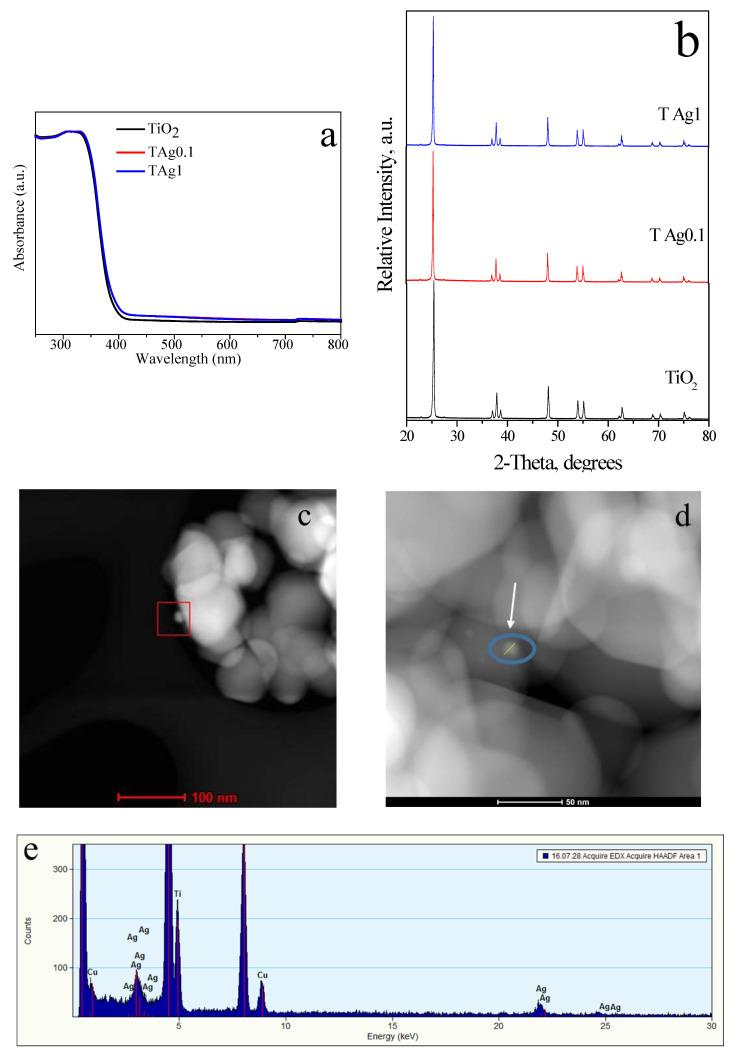
(**a**) DRS and (**b**) DRX of TiO_2_ and TiO_2_ decorated with NPs; TEM of (**c**) TAg0.1 and (**d**) TAg1; (**e**) EDS of TAg0.1.

Figure 9 shows DRS, XRD, TEM, and EDX characterizations for TiO_2_-AgNPs with 0.1 wt% of P103 (TAg0.1) and for TiO_2_-AgNPs with 1 wt% of P103 (TAg1). On the one hand, Figure 9a exhibits the optical response of TiO_2_ composites. All materials displayed strong absorption at wavelengths shorter than 400 nm, attributed to the absorption of the TiO_2_ support. In addition, decorated materials demonstrate a slight increase in absorption, from 400 to 700 nm, due to the LSPR effect of AgNPs [54]. Figure 9b shows the diffractograms of the prepared TiO_2_ composites where the observed reflections (25.3°, 36.9°, 48.1°, 53.9°, and 55.1°) are characteristic of the anatase-TiO_2_ phase, this in good agreement with the JCPDS card No. 21-1272 [29,54]. No discernible differences were noticed between decorated and undecorated materials. On the other hand, Figure 9c,d illustrates the TEM images of the composites where AgNPs (~15 nm) decorating the TiO_2_ surface were visualized. Additionally, elemental silver (Figure 9e) is identified through EDS exhibiting a peak around 3 keV [29,55,56]. The absence of carbon suggests that the polymer (0.1 and 1 wt%) is removed by heat treatment.

**Figure 10:**
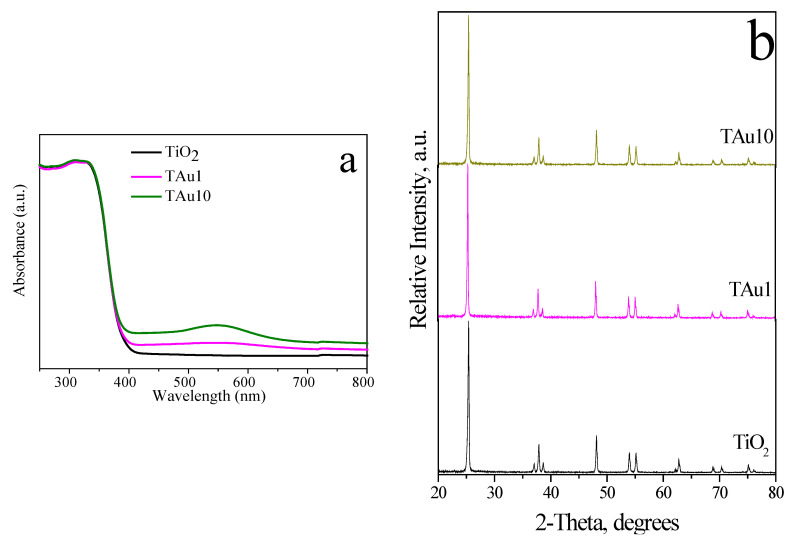

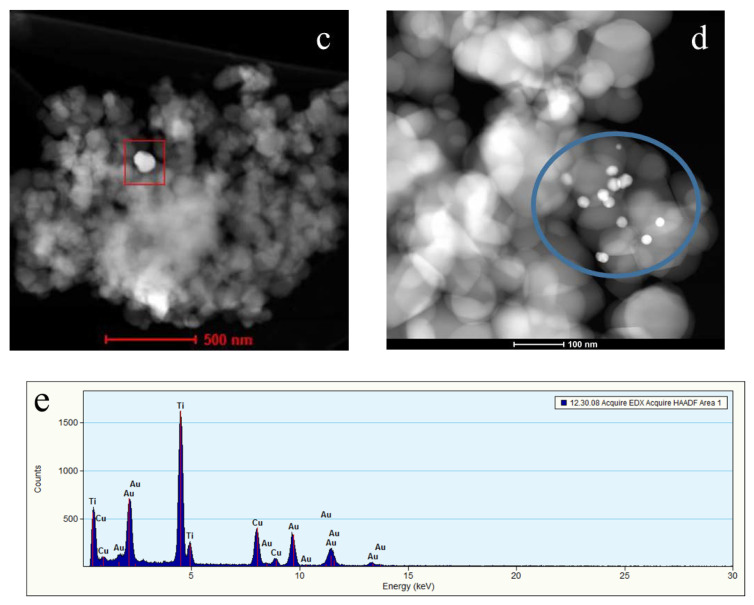
(**a**) DRS and (**b**) DRX of TiO_2_ and TiO_2_ decorated with NPs, (**c**) TEM of TAu1, (**d**) TEM of TAu10, and (**e**) EDS of TAu1.

The optical properties of TiO_2_-Au composites can be investigated by diffuse reflectance UV-Vis spectroscopy, because the LSPR peaks of AuNPs are very sensitive to size and aggregation [57]. Decorated materials with AuNPs demonstrate an increase in light absorption (Figure 10a). TiO_2_-AuNPs with 1 wt% of P103 (TAu1) exhibit a very broad band, suggesting NPs aggregation. However, TiO_2_-AuNPs with 10 wt% of P103 (TAu10) present a well-defined band with an absorption peak located at ~540 nm, the latter proposing better particle distribution on the support. Figure 10b illustrates the XRD powder patterns of TiO_2_ composites. Similar to Ag-TiO_2_ composites, the diffraction peaks are characteristic of the anatase phase; the diffraction patterns remained unchanged, it is concluded that neither AgNPs nor AuNPs alter the TiO_2_ crystal structure [57]. Zhang et al. [58] suggest that the sizes of the AuNPs are too small and the gold content in the composite is lower than the XRD detection limit. Therefore, TEM micrographs are performed, and the presence of spherical AuNPs is observed (Figure 10c,d). Here, TAu1 reveals a large nanoparticle (90 nm), while TAu10 exhibits spherical-shaped particles with a mean diameter of 21 nm. This mean size is larger than those reported by DLS (Figure 8d), proposing the agglomeration of NPs during calcination. Finally, elemental gold is identified through EDS, producing strong signals near 2, 9.5, and 11.5 keV (Figure 10e) [55,59].

**Figure 11:**
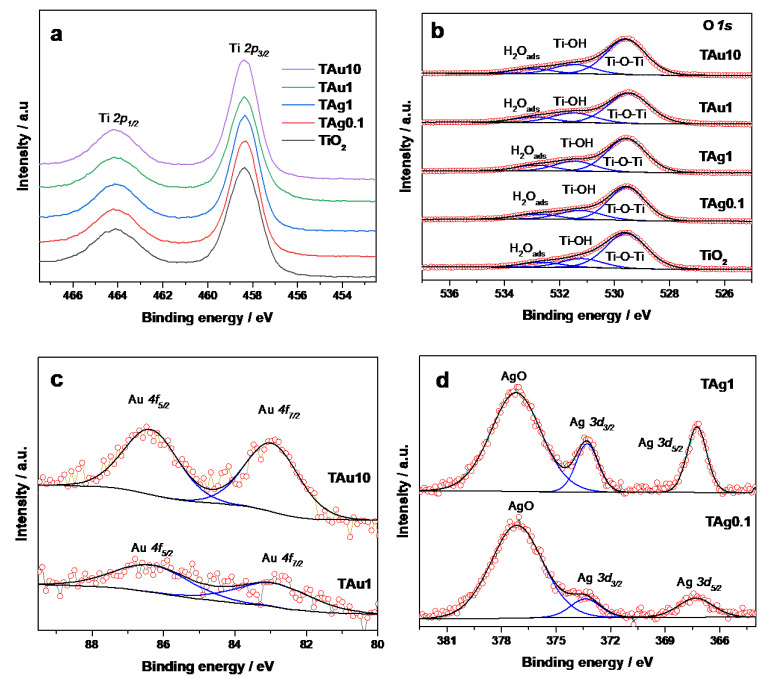
XPS spectra results of (**a**) Ti 2p zone of all samples; (**b**) O 1s zone of all samples; (**c**) Au 4f zone of TAu1 and TAu10 samples; and (**d**) Ag 3d zone of TAg0.1 and TAg1 samples.

The physical-chemical properties of the photocatalysts were also analyzed using XPS. As expected, analysis of the Ti 2p signal (Figure 11a) in all samples provides evidence of a Ti(IV) chemical state (Ti 2p3/2 and Ti 2p1/2 binding energy peaking at 458.4 and 464.1 eV for all samples) characteristic of titanium oxides [60,61]. Following the analysis carried out in this work, three characteristic peaks are observed in the O 1s spectra (Figure 11b) for all samples. The band centered at 532.7 eV is attributed to the adsorbed water, whereas the peak centered al 531.2 eV corresponds to the hydroxyl species adsorbed on the TiO_2_ surface (Ti-OH). Finally, peak binding energy at 529.6 eV is related to the lattice oxygen of TiO_2_ or the metal-oxygen bond (Ti-O). In the event of a significant modification of the titanium dioxide structure, the intensity of the peak associated to the crystal lattice oxygen (529.6 eV) would decrease due to the oxygen vacancies generated by the noble metal incorporation into the support semiconductor structure as previously reported by [62,63,64]. However, the titanium and oxygen XPS results display rather small variations among the samples (Pure TiO_2_ vs. Au- or Ag-modified TiO_2_), revealing similar chemical properties of the TiO_2_ component in the materials, indicating that the Au or Ag incorporation on Titania, carried out in this work, does not modify the TiO_2_ structure, and therefore we obtain only a surface modification. In addition, the oxidation state of the noble metals was studied with XPS (Figure 11c,d). In the case of Au results, constant values of the characteristic doublet of Au(0) metallic state, signals at 83.0 ± 0.1 eV (Au 4f7/2) and 86.4 ± 0.1 eV (Au 4f5/2), were obtained for TAu1 and TAu10 samples [62,63]. Meanwhile, the TAg0.1 and TAg1 spectra results displayed values at 367.3 ± 0.1 eV (Ag 3d5/2) and 373.3 ± 0.1 eV (Ag 3d3/2), which correspond to the Ag(0) oxidation state, while the peak signal value at 377.3 ± 0.1 eV corresponds to the Ag(II) oxidation states [65].

### 2.4. TiO_2_-AgNPs and TiO_2_-AuNPs Photocatalytic Tests

**Figure 12:**
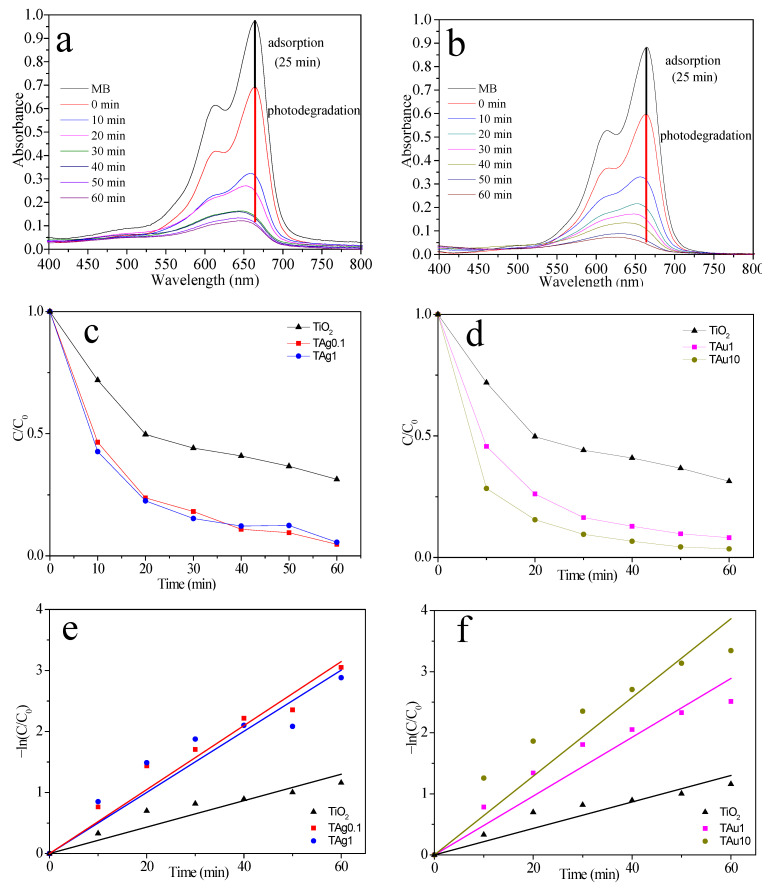
UV–Vis absorption spectra showing changes in the intensity of the MB dye under exposure to ultraviolet and visible-light: (**a**) TAg0.1; (**b**) TAu10. Variation of the photocatalytic degradation rate of MB dye on TiO_2_ and decorated composites with time of irradiation (**c**,**d**). The pseudo first order kinetics of the degradation of MB dye on TiO_2_ and decorated samples (**e**,**f**).

The photocatalytic activity of TiO_2_ composites is analyzed by (1) adsorption and (2) photodegradation of MB, through UV-Vis spectroscopy (Figure 12a,b). Photodegradation occurs through two mechanisms: (1) molecule breakdown (0–10 min), in which absorption spectra show no change in wavelength of the maximum absorbance peak (664 nm), and (2) the N-demethylation process (20–60 min), when the absorption spectra exhibit a slight blueshift, suggesting considerable photodegradation activity [58]. Figure 12c,d demonstrates the photocatalytic performance for bare TiO_2_, silver and gold composites, where C_0_ is the initial concentration without light irradiation and C is the concentration of MB varied over time. The MB degradation rates are graphically shown in Figure 12e,f. Degradation reaction kinetics follow a pseudo-first order reaction. The rate constant for bare TiO_2_ is k = 0.02167 min^−1^, TAg0.1 k = 0.05242 min^−1^, TAg1 k = 0.05009 min^−1^, TAu1 k = 0.04814 min^−1^, and TAu10 k = 0.06443 min^−1^. It is obvious that k of all samples is larger than that of bare TiO_2_. Other authors have obtained similar results [66,67]. The TAu10 photocatalyst has the best photocatalytic activity for degradation of the MB aqueous solution. It is evident that the amount of polymer used during the synthesis of NPs modifies its size, and consequently its efficiency as a catalyst.

**Figure 13:**
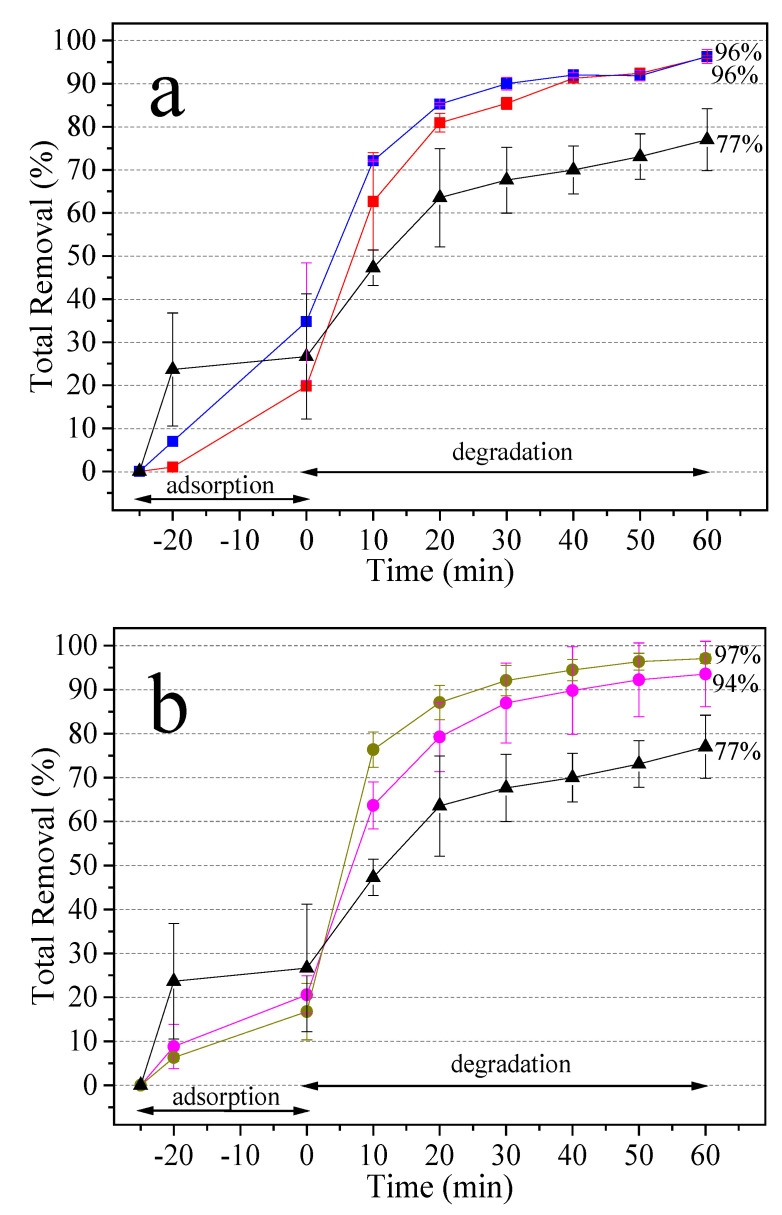
Total removal of MB (%): (**a**) (■) TAg0.1 and (■) TAg1; (**b**) (●) TAu1 and (●) TAu10. Both graphs use (▲) TiO_2_ as a reference.

Figure 13 shows MB removal by the produced composites. Figure 13a exhibits Ag-decorated composites (TAg0.1 and TAg1) and bare TiO_2_ samples. MB oxidizes via photo-reactivity, as evidenced by the increase in degradation as a function of irradiation time. The decorated materials exhibit 96% total removal, demonstrating that NPs affect the result in the process. Figure 13b presents Au decorated composites (TAu1 and TAu10) and bare TiO_2_ samples. Again, and as expected, decorated materials display high degradation in the photocatalytic process.

Table 1 presents the textural analysis results. Regarding the specific surface area, no substantial differences were observed between bare TiO_2_ and the composites. On the other side, a slight pore-size decrease was detected after the introduction of noble metal nanoparticles, which was attributed to TiO_2_ pore blockage by AuNPs or AgNPs [54,68]. Based on the results, it is not feasible to assign a considerable surface area effect of NPs to the decorated composites.

Bare TiO_2_ and the composites were characterized by diffuse reflectance spectroscopy in order to determine their bandgap energy (see Table 1), which was calculated by the Tauc plot and the Kubelka-Munk function [67,69]. For decorated composites, the band gap energy was shifted to a slightly lower level than anatase-TiO_2_, which might be the result of the size-dependent quantum confinement effect [68]. Due to the small amount of nanoparticles incorporated onto the TiO_2_ surface, the nanoparticles do not considerably influence the UV-Vis absorption spectra (Figure 9a and Figure 10a); therefore, no considerable change in the bandgap of the materials is observed. However, the homogeneous distribution of the appropriate amount of metallic nanoparticles on the titania surface is a fundamental factor in optimizing the photocatalytic properties [66].

In the degradation stage, TAg0.1 and TAu10 composites reached the highest percentage of dye degradation, that is, 76%, and 80%, respectively, although the material with the highest percentage of total removal was TAu10 (97%).

Different authors suggest that Au Nps and Ag NPs loaded on semiconductors can absorb visible light, resulting in the collective oscillation of the electrons (hot electrons). These hot electrons are injected into the semiconductor conduction band (CB) through the interface between metal and semiconductor, thus, facilitating photogenerated carrier separation and consequently reducing the pair recombination of electrons and holes [23,24,36,54,70]. Singh et al., propose that Au nanoparticles facilitate the formation of superoxide radicals (•O_2_^−^) from oxygen molecules. On the other hand, water molecules interacted with holes and they were converted into hydroxyl radicals (•OH). These reactive species were responsible for the degradation of the MB dye [26]. Matsunami et al. suggest that the degradation of MB is carried out by the processes of N-demethylation and the cleavage of C-N and C-S bonds [71].

Similar results were obtained by Messih et al. [66], who achieved 95% degradation of the MB pollutant model using Ag/TiO_2_ nanocomposites synthesized through “green” methods. The incorporation of silver on the surface of titania increased its photocatalytic reactivity under UV radiation and sunlight, exhibiting better performance than pure titania. On the other hand, Perera et al. [12] fabricated Au/TiO_2_ nanocomposites by means of a green chemical approach. Decoration of the TiO_2_ surface with AuNPs significantly increases the MB adsorption capacity of the catalyst, and at the same time increases the photocatalytic degradation rate constant. Researchers agree that there is a need to migrate from classical chemical reduction methods to novel, easy, and environmentally friendly mechanisms to prepare decorated composites. It has been shown that surface decoration of TiO_2_ with metallic NPs, such as silver and gold, increases photocatalytic efficiency under UV irradiation and even on employing visible radiation. The results are promising with model molecules, which motivates further study, in that it is envisioned that these nanostructures can be utilized in various applications, such as in environmental remediation, solar cells, and energy production [12,29,66].

## 3. Materials and Methods

### 3.1. Materials

The following materials were used: Tetrachloroauric (III) acid trihydrate (HAuCl_4_∙3H_2_O, Aldrich, 99.94%), silver nitrate (AgNO_3_, Aldrich, 99.9999%), triblock copolymer Pluronic P103 (PEO_17_-PPO_60_-PEO_17_, BASF), sodium borohydride (NaBH_4_, Aldrich, ≥98.0%), titanium dioxide (TiO_2_, J. T. Baker, >99%), and methylene blue (MB) (Hycel, IC 52015 indicator). The reagents were used directly, without further purification.

### 3.2. Synthesis of Ag Nanoparticles

Aqueous solutions of the triblock copolymer Pluronic P103 were prepared at different concentrations (0.0, 0.01, 0.1, and 1.0 wt%). Samples were placed in a water bath at 30 °C for 12 h to ensure stabilization of the different polymeric structures.

The aqueous solutions of the copolymer and the aqueous solution of AgNO_3_ (1 mM) were stored in glass vials. A fresh NaBH_4_ (7 mM) solution was used as a reducing agent. The mixture ratio of AgNO_3_, NaBH_4_ solution, and polymer solution was 1:4:4, respectively [8]. The reactions were carried out at 30 °C for 4 h in the presence of visible light. Finally, the colloidal solutions were washed with water and centrifuged at 19,000 rpm for 30 min at room temperature. This procedure was repeated three times.

### 3.3. Synthesis of Au Nanoparticles

The synthesis of AuNPs was performed following the Sakai methodology with some adaptations [50]. Aqueous solutions of the triblock copolymer Pluronic P103 were prepared at different concentrations (0.5, 1.0, and 10 wt%). Samples were placed in a water bath at 30 °C for 12 h to ensure stabilization of the different polymeric structures. The mixture ratio of HAuCl_4_∙3H_2_O (2 mM) and the polymer solution was 1:9. In this case, the triblock copolymer acted as a reducing and stabilizing agent. Reactions were carried out at 30 °C for 4 h in the presence of visible light. Finally, the colloidal solutions were purified by washing cycles with water and centrifuging at 19,000 rpm for 30 min at room temperature. This procedure was repeated three times.

### 3.4. Synthesis of the TiO_2_-AgNPs and TiO_2_-AuNPs Composites

The synthesis of TiO_2_-AgNPs (TAgX) and TiO_2_-AuNPs (TAuX), where X represents the Pluronic P103 concentration, was achieved using 0.5 g of TiO_2_ with 10 mL of AgNPs or 30 mL of AuNPs, respectively. The distinct NPs amounts (10 vs. 30 mL of Ag and Au, respectively) were determined based on the screening of the catalytic experiments since the higher activity of AgNPs over AuNPs is well-recognized. The suspensions were stirred in an ultrasonic bath for 5 min to ensure high dispersion of the NPs. Subsequently, they were dried in an oven at 80 °C and were finally calcined in a muffle at 500 °C for 30 min. Additionally, a reference sample, i.e., bare TiO_2,_ was used under the same conditions to compare its behavior with the decorated materials.

### 3.5. Photocatalytic Activity Experiments

The photocatalytic activity study was performed using 0.015 g of TiO_2_-AgNPs or TiO_2_-AuNPs composite powder dispersed in 50 mL of MB (10 ppm). These suspensions were maintained under dark conditions to achieve an adsorption-desorption equilibrium prior to irradiation. After 25 min, the photocatalytic systems were irradiated with ultraviolet and visible light (OSRAM, 15 W, 365–465 nm, OSRAM LED, 30 W, 450–750 nm) for 60 min. To study the photocatalytic performance, aliquots of the suspensions were removed every 10 min. In this manner, it was possible to monitor the absorption intensity around 664 nm by UV-Vis spectroscopy. The initial measured pH of the suspension was 6.5, and the pH was allowed to vary freely during the reaction.

Since the absorbance values are directly proportional to dye concentration, the adsorption on the catalyst surface (Equation (1)) and the photocatalytic degradation (Equation (2)) allowed calculating the total dye removal (Equation (3)) [11,22,25,66,72,73].

(1) $$\%\;\mathrm{adsorption}=\left(1-\;\frac{A_{0}}{A_{\mathrm{AM}}}\right)\times 100\%$$

(2) $$\%\;\mathrm{degradation}=\left(1-\;\frac{A_{60}}{A_{0}}\right)\times 100\%$$

(3) $$\%\;\mathrm{total}\;\mathrm{removal}=\left(\;1-\frac{A_{60}}{A_{\mathrm{AM}}}\right)\times 100\%$$

with A_AM_ maximum absorbance of MB, A_0_ maximum absorbance at t = 0 min, i.e., at the end of the adsorption on the catalyst surface, and A_60_ maximum absorbance at t = 60 min.

### 3.6. Materials Characterization

AgNPs and AuNPs were characterized by UV-Vis spectroscopy and Dynamic Light Scattering (DLS). These techniques were employed due to their simplicity, sensitivity, selectivity, and short measurement time [74]. The optical properties of the colloidal solutions were studied and analyzed by UV-Vis spectroscopy using a GENESYS 10S UV-Visible spectrometer (Thermo Scientific, Waltham, MA, USA) at 30 °C. A quartz cell with an optical path of 1 cm was utilized. The sizes of the NPs were determined by DLS using a Zetasizer 4000 (Malvern-Panalytical, Worcestershire, UK). The light source was a 5-mW He-Ne laser at 632.8 nm. The scattering angle was maintained at 90° and the measurement time was 120 s. The sizes and shapes of the NPs were determined by Transmission Electron Microscopy (TEM) analyses, using a JEOL-JEM-2010 (JEOL, Tokyo, Japan) in conventional transmission mode, operating at 80 kV. Samples were prepared by placing a drop of the solution on a carbon-coated Cu grid before air drying the samples.

The decorated material was characterized by DRS, XRD, BET surface area measurements, TEM and EDS. For diffuse reflectance spectroscopy (DRS), a UV Vis-NIR spectrophotometer (Cary 5000) equipped with an integrating sphere from Agilent Technologies was utilized; with KBr as the reference sample. The X-ray diffraction patterns were obtained in an XRD Bruker D8 Advance diffractometer with an X-ray generator of Cu (K_λ_ = 0.15406 nm) and a NaI detector with a scan rate of 0.02° min^−1^, 2*θ* range from 5° to 80°. Composites were characterized by high angle annular dark field scanning transmission electron microscopy (HAADF-STEM) using an FEI TECNAI F30 (FEI, MA, USA) Transmission Electron Microscope (FEG-TEM 300 kV). Samples were dispersed in 2-propanol by sonication and then dropped on gold coated holey carbon grids for observation. Line-scan profile energy-dispersive X-ray spectroscopy (EDS) measurements were obtained with an EDAX detector system. The specific surface area and pore sizes were calculated by the Brunauer–Emmett–Teller theory. Samples were degassed at 80 °C for 360 min, using a Micromeritics surface area and a pore-size analyzer, model ASAP2020. The XPS spectra of the samples were recorded using a SPECS^®^ spectrometer with a PHOIBOS^®^ 150 WAL hemispherical energy analyzer with angular resolution (<0.5 degrees), equipped with sources: an XR 50 Al-X-ray and a μ-FOCUS 500 X-ray monochromator (Alexcitation line). Samples were first degassed at 10–5 mbar in the pretreatment chamber before being transferred to the analysis chamber, where residual pressure was maintained at below 5 × 10^−9^ mbar during data acquisition. The binding energies (BE) were referenced to the C 1s peak (284.6 eV) to account for charging effects. Surface chemical compositions were estimated from XP-spectra by calculating the integral of each peak after subtraction of the “S-shaped” Shirley-type background [75] using the appropriate experimental sensitivity factors and CASA-XPS (version 2.3.15) software.

## 4. Conclusions

It is well known that the incorporation of metallic NPs onto the surface of a semicon-ductor, as in TiO_2_, improves its photocatalytic properties. However, there is a wide variety of methodologies for synthesizing metallic NPs.

The simple synthesis of AgNPs and AuNPs with Pluronic P103 copolymer is reported. AgNPs synthesis considers three reagents, i.e., an AgNO_3_ solution, a NaBH_4_ solution as a reductant, and a triblock copolymer solution (Pluronic P103) at different concentrations as a stabilizer. However, AuNPs synthesis considers only two reagents: an HAuCl_4_:3H_2_O solution and a triblock copolymer solution, which act as a reductant and stabilizer. Consequently, the method is considered environmentally friendly due to the amount and type of reagents used, together with the fact that the copolymer presents high biocompatibility and low bioaccumulation. Additionally, the number of steps was decreased, thus diminishing the time and residues compared to conventional synthesis procedures. It is worth emphasizing that the results herein presented evidence that copolymer concentration modifies the size of NPs. As the polymer concentration increases, the size of the NPs increases. However, by further increasing the amount of polymer, it is possible to generate soft templates where notably smaller particle sizes are generated. The synthesized NPs were utilized to decorate the TiO_2_ surface and were evaluated by MB photodegradation. The evaluation results demonstrated that the copolymer concentration along with its effect on the size and shape of the NPs, influence MB photodegradation, increasing its photocatalytic activity up to 20% compared to bare TiO_2_. By simple methods such as the one presented here, it is possible to obtain promising materials in various fields, including photocatalysis. For this reason, the MB dye was used as a probe molecule to demonstrate that the composites were able to improve anatase-TiO_2_ activity.

## Figures and Tables

**Table 1:** BET surface area, band gap, adsorption, degradation, and total removal percentages of MB in TiO_2_ composites.

Composites	S_BET_ ^a^ (m²/g)	Pore Size (nm)	Band Gap ^b^	Band Gap ^c^ (eV)	Adsorption (%)	Degradation (%)	Total Removal (%)
TAg0.1	9.0	1.29	3.14	3.21	20	76	96
TAg1	13.2	1.30	3.14	3.20	35	61	96
TAu1	8.3	1.08	3.14	3.21	21	73	94
TAu10	9.4	1.13	3.16	3.19	17	80	97
TiO_2_	9.1	1.33	3.18	3.22	27	50	77

^a^ S_BET_, BET surface areas calculated by the adsorption/desorption isotherm. ^b^ Band gap was calculated by the Tauc Plot. ^c^ Band gap was calculated by the Kubelka-Munk function.

## Data Availability

Data are contained within the article.
